# A combined hypoxia and immune gene signature for predicting survival and risk stratification in triple-negative breast cancer

**DOI:** 10.18632/aging.203360

**Published:** 2021-08-02

**Authors:** Xia Yang, Xin Weng, Yajie Yang, Meng Zhang, Yingjie Xiu, Wenfeng Peng, Xuhui Liao, Meiquan Xu, Yanhua Sun, Xia Liu

**Affiliations:** 1 Department of Pathology, The First Affiliated Hospital of Shen Zhen University, Shenzhen, China; 2 Department of Pathology, Shenzhen Second People’s Hospital, Shenzhen, China

**Keywords:** triple-negative breast cancer, risk stratification, hypoxia, immune, survival

## Abstract

Background: Increasing evidence showed that the clinical significance of the interaction between hypoxia and immune status in tumor microenvironment. However, reliable biomarkers based on the hypoxia and immune status in triple-negative breast cancer (TNBC) have not been well established. This study aimed to explore a gene signature based on the hypoxia and immune status for predicting prognosis, risk stratification, and individual treatment in TNBC.

Methods: Hypoxia-related genes (HRGs) and Immune-related genes (IRGs) were identified using the weighted gene co-expression network analysis (WGCNA) method and the single-sample gene set enrichment analysis (ssGSEA Z-score) with the transcriptomic profiles from Molecular Taxonomy of Breast Cancer International Consortium (METABRIC) cohort. Then, prognostic hypoxia and immune based genes were identified in TNBC patients from the METABRIC (*N* = 221), The Cancer Genome Atlas (TCGA) (*N* = 142), and GSE58812 (*N* = 107) using univariate cox regression model. A robust hypoxia-immune based gene signature for prognosis was constructed using the least absolute shrinkage and selection operator (LASSO) method. Based on the cross-cohort prognostic hypoxia–immune related gene signature, a comprehensive index of hypoxia and immune was developed and two risk groups with distinct hypoxia–immune status were identified. The prognosis value, hypoxia and immune status, and therapeutic response in different risk groups were analyzed. Furthermore, a nomogram was constructed to predict the prognosis for individual patients, and an independent cohort from the gene expression omnibus (GEO) database was used for external validation.

Results: Six cross-cohort prognostic hypoxia–immune related genes were identified to establish the comprehensive index of hypoxia and immune. Then, patients were clustered into high- and low-risk groups based on the hypoxia–immune status. Patients in the high-risk group showed poorer prognoses to their low-risk counterparts, and the nomogram we constructed yielded favorable performance to predict survival and risk stratification. Besides, the high-risk group had a higher expression of hypoxia-related genes and correlated with hypoxia status in tumor microenvironment. The high-risk group had lower fractions of activated immune cells, and exhibited lower expression of immune checkpoint markers. Furthermore, the ratio of complete response (CR) was greatly declined, and the ratio of breast cancer related events were significantly elevated in the high-risk group.

Conclusion: The hypoxia–immune based gene signature we constructed for predicting prognosis was developed and validated, which may contribute to the optimization of risk stratification for prognosis and personalized treatment in TNBC patients.

## INTRODUCTION

Triple-negative breast cancer (TNBC) is a special subtype of breast cancer that lacks the expression of ER (estrogen receptor), PR (progesterone receptor), and HER2 (human epidermal growth factor receptor 2). TNBC is characterized by high aggression and invasiveness that exhibit the most malignant biological behavior and the worst clinical outcome [1]. For the treatment of TNBC, neither endocrine therapy nor targeted therapy for HER2 could be applied in clinical practice [2]. Traditional therapeutic methods like surgery and systemic chemotherapy are still the first-line treatment for TNBC. Therefore, it is urgent to understand the biological and immunological profiles of TNBC to develop novel effective therapeutic strategies.

According to genome-wide expression profile, TNBC has been classified into six distinct molecular subtypes, including basal-like 1 (BL1), basal-like 2 (BL2), luminal androgen receptor (LAR), immunomodulatory (IM), mesenchymal (M), and mesenchymal stem-like (MSL) groups [3]. Besides, Burstein et al. identified four TNBC subgroups based on multi-omics genomic profiling, which clustered into Luminal/Androgen Receptor, Mesenchymal, Basal-Like Immune Suppressed, and Basal-Like Immune Activated groups [4]. Bareche et al. observed a higher expression level of immune signatures and checkpoint inhibitor genes in the IM subtype, which implied a better prognosis [5]. These efforts indicated that the heterogeneous immune profile in tumor microenvironment, and immunotherapies might be practical in some specific subtypes of TNBC.

Close attention has been given to the progression of immunotherapy in TNBC. Several immune-checkpoint inhibitors, including anti-cytotoxic T-lymphocyte-associated protein 4 (anti-CTLA-4), anti-programmed death-1 (anti-PD1), and anti-PD1 ligand (anti-PD-L1) monoclonal antibodies have been applied for selected advanced TNBC, which present favorable prognostic value in clinical trials [6–9]. The cooperation between tumor cells and extracellular microenvironment has been proved as an important indicator for therapeutic response and prognosis of TNBC [10–14]. Increasing evidence showed that the interaction between hypoxia and immune status in tumor microenvironment promotes the proliferation, migration, and invasion of TNBC [15–18].

Hypoxia is an intrinsic feature of solid tumors due to the imbalance between the proliferation rate of tumor cells and insufficient nutrient supply of vascular [19]. Increasing studies have recognized the important roles played by hypoxia in driving tumor immune suppression and immune escape. For instance, hypoxia increases the expression level of immunosuppressive cytokines (e.g., PD-1) and suppressive cells [e.g., regulatory T cells (Tregs) and myeloid-derived suppressor cells (MDSCs)], which in turn impede immune effector cells and induce immune escape [16]. Moreover, hypoxia triggers the IL-1β/IL1R1 signaling that leads to proliferative and invasive response of TNBC cells and promotes an aggressive feature of cancer-associated fibroblasts (CAFs) in TNBC [20]. Given that the interdependence between hypoxia and immune status in tumor microenvironment might affect the immune activity, therapeutic response and prognosis in TNBC, a comprehensive analysis of hypoxia and immune status might have promising prognostic value, and offer additional introspection and improvement for transformation studies and therapeutic decisions in TNBC.

In this study, by performing a comprehensive bioinformatics analysis based on cross-public datasets, we aimed to establish and substantiate a combined hypoxia and immune-related gene signature to predict prognosis, risk stratification, and therapeutic response in TNBC patients.

## MATERIALS AND METHODS

### Data acquisition and preparation

**Figure 1:**
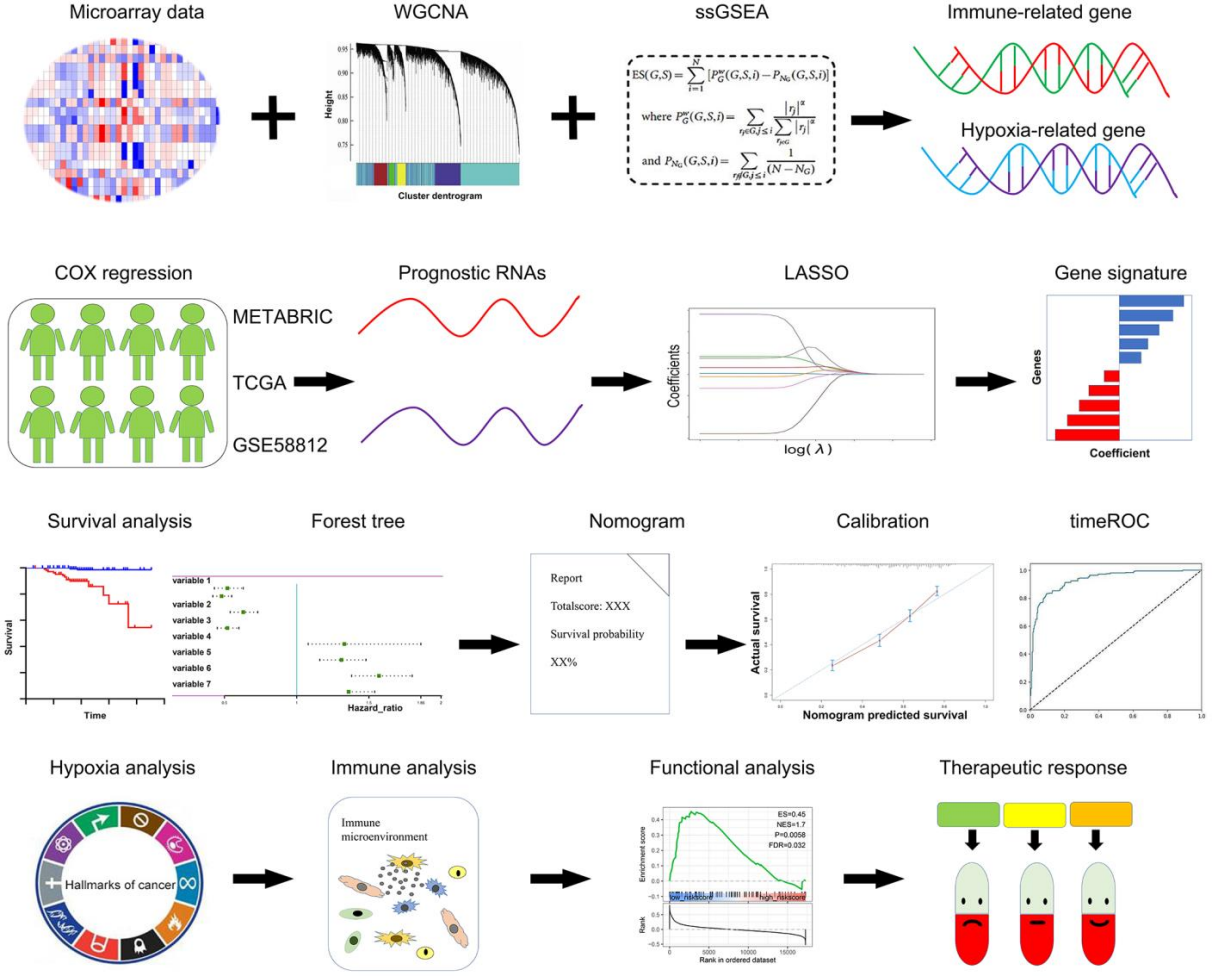
**Schematic diagram of this study.** A panel of prognostic hypoxia-related and immune-related genes were determined from the METABRIC, TCGA, and GSE58812 datasets. A comprehensive hypoxia and immune related genes were constituted using the LASSO regression model. The prognostic value, hypoxia and immune status, and therapeutic response were further validated in multiple cohorts.

The Schematic diagram is depicted in Figure 1. TNBC patients with clinical features and survival data across different platforms were enrolled in this study. The microarray dataset GSE58812 (*N* = 107) were downloaded from GEO (http://www.ncbi.nlm.nih.gov/geo/) [21], the normalized RNA-Seq data of 142 TNBC samples were procured from The Cancer Genome Atlas (TCGA) (https://portal.gdc.cancer.gov/repository), and the expression profiles of the Molecular Taxonomy of Breast Cancer International Consortium (METABRIC) TNBC dataset (*N* = 221) was obtained from cBioportal (http://www.cbioportal.org/) [22].

R package ‘limma’ was applied for gene expression normalization [23]. All transcriptomic data contained in this study were normalized. This study strictly followed the acquirement procedures of the METABRIC, TCGA, and GEO datasets. This research also complied with the instruction of the Declaration of Helsinki.

### Identification of hypoxia status and hypoxia-related genes

The hallmark gene sets of hypoxia which including 200 genes were obtained from the Molecular Signatures Data base (MSigDB) (https://www.gsea-msigdb.org/gsea/msigdb/). First, we evaluated the hypoxia status in TNBC from the METABRIC dataset by the ssGSEA algorithm (R package ‘gsva’) [24]. Then, we established a scale-free co-expression network and determine the hypoxia-related module by the package ‘wgcna’ [25]. The interaction between distinctive genes with hypoxia ssGSEA score were quantified by Gene significance (GS), and the correlation of gene expression profiles and module eigengenes were represented by module membership (MM). With a threshold of GS *p* < 0.05, 840 candidate genes from the ‘pink module’ were selected.

### Identification of the immune status and immune-associated genes

For the IRGs, 22 immune signatures were measured for their enrichment levels in respective TNBC cases by ssGSEA score [26, 27]. Patients were hierarchically clustered into three groups (high, median and low immune group) based on the ssGSEA score. Differential Expression Genes (DEGs) between high and low immune groups were identified by the “limma” package. Furthermore, genes with |log2 value (FC)| >1 and *p* < 0.05 after adjusting for FDR were considered as the immune-related DEGs. Finally, 1793 DEGs were identified from the above analyses.

### Construction and verification of the prognostic value of hypoxia and immune related gene signatures

In total, 788 HRGs and 1175 IRGs were selected in the cross-cohort. Next, we analyzed the prognostic significance of these HRGs and IRGs by univariate Cox regression using the R package ‘survival’. Subsequently, we selected the most robust prognostic gene signatures in LASSO regression model using the R package ‘glmet’ [28]. Then, a hypoxia-immune related risk score (HIRS) was calculated by the corresponding coefficients of selected signatures. The HIRS formula was established as follows:

Score = Σ*i Coefficient* (*mRNA*) × *Expression* (*mRNA*) [29].

According to the median value of HIRS, patients were divided into low-risk and high-risk groups.

### Tumor microenvironment analysis

CIBERSORT was performed to analyze the divergent immunocyte infiltrating proportion between low-risk and high-risk groups in conformity with LM22 signatures with 1000 permutations [30]. The immune-related efficiency and fibroblasts were estimated using the ‘MCPcounter’ package [31]. immune and stromal components which reflect by immune and stromal scores were estimated using the ‘estimate’ package [32]. Moreover, the expression of key hypoxia and immune profiles between different risk groups were analyzed.

### Functional study and therapeutic response

Gene set enrichment analysis (GSEA) [33] was performed to investigate the signaling enrichment between different risk groups using prognosis index with Clusterprofile package. The FDR *q* < 0.25 and *P* < 0.05 was considered statistical significance. 107 patients with survival information from GSE103091 cohort were obtained to validate the indicative significance of the hypoxia and immune gene signature. The data from GSE18864 and GSE90505 cohorts were obtained to analyze the indicative role of the hypoxia and immune gene signature for therapeutic response. Furthermore, a webtool GSCALite was applied to analyze the relationship between the expression profile of hypoxia-immune gene signature and IC50 data of different molecules in breast cancer cell lines [34].

### Statistical analysis

The cross-cohort prognostic HRGs and IRGs were identified by Univariate Cox regression with a cutoff value of *P* < 0.1. Vital prognostic hypoxia and immune related genes were selected by the LASSO regression model. Multivariate Cox regression including HIRS and clinical characteristics was performed using the ‘survival’ package. The survival of different risk groups was evaluated using the Kaplan–Meier survival analysis with the log-rank test. A nomogram was plotted using the R package ‘rms’ to predict the prognosis for individual patients [35]. Time-dependent receiver operator characteristic (ROC) analyses were conducted to measure the predictive power of the nomogram using the ‘time-ROC’ package [36], and the areas under the curve (AUC) of all variables were compared. The divergence between immune cell fragments was estimated by the Wilcoxon test. HIRS-related analysis was estimated by Spearman’s correlation test. Statistical analyses were applied using R software (Version 4.0.4). A two-tailed *P* < 0.05 was considered statistical significance.

### Data availability statement

This study is based on public datasets, which obtained from the Cancer Genome Atlas Program (TCGA), the cBio Cancer Genomics Portal (cBioportal), and Gene Expression Omnibus (GEO).

## RESULTS

### Identification of gene signature related to hypoxia in TNBC

**Figure 2:**
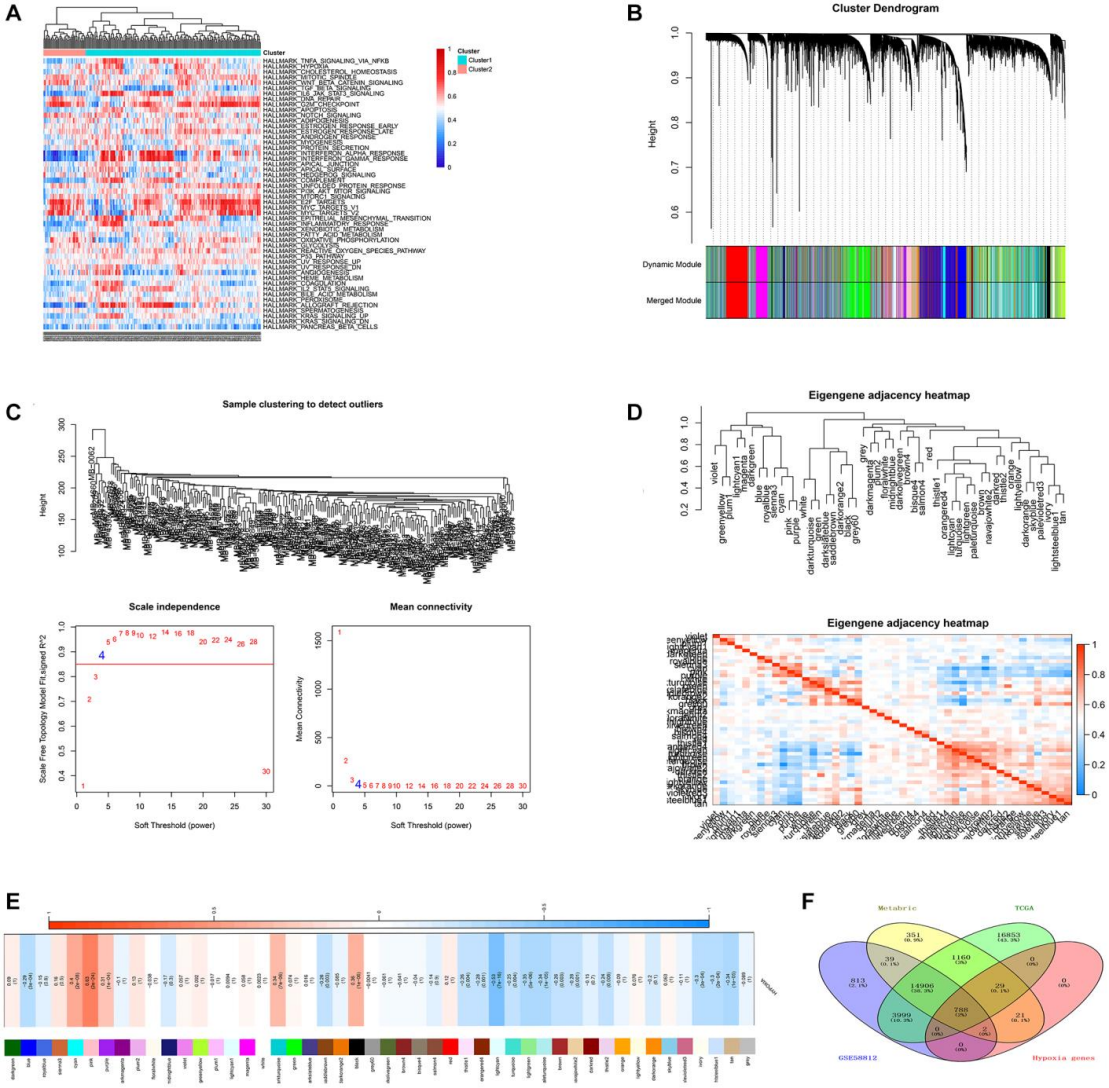
**Identification of potential HRGs in TNBC.** (**A**) Hypoxia ssGSEA scores were estimated in the METABRIC cohort. (**B**) WGCNA was applied with whole-transcriptome profiling data and hypoxia ssGSEA Z-scores. (**C**) The optimal soft threshold to confirm a scale free co-expression network. (**D**) A total of 47 non-grey modules were identified. (**E**) The pink module depicted the highest correlation (*r* = 0.64, *p* = 2e−24) with hypoxia. (**F**) Venn diagram suggested 788 hypoxia related genes in the three cohorts.

Based on the ssGSEA method and cancer hallmarks from MsigDB dataset, we calculated the hypoxia ssGSEA Zscore of TNBC patients from the METABRIC dataset (Figure 2A). Then, with transcriptomic profiles and hypoxia ssGSEA Z-scores in the METABRIC dataset, WGCNA was applied to screen for hypoxia related candidates (Figure 2B). the optimal soft threshold was determined with a power of β = 4 (Figure 2C), 47 modules were established (Figure 2D), and the pink module showed the highest correlated with hypoxia (Figure 2E), 840 promising candidates were identified from the pink model. Finally, 788 promising candidates related to hypoxia were screened in the three datasets (Figure 2F).

### Identification of gene signature related to immune in TNBC

**Figure 3:**
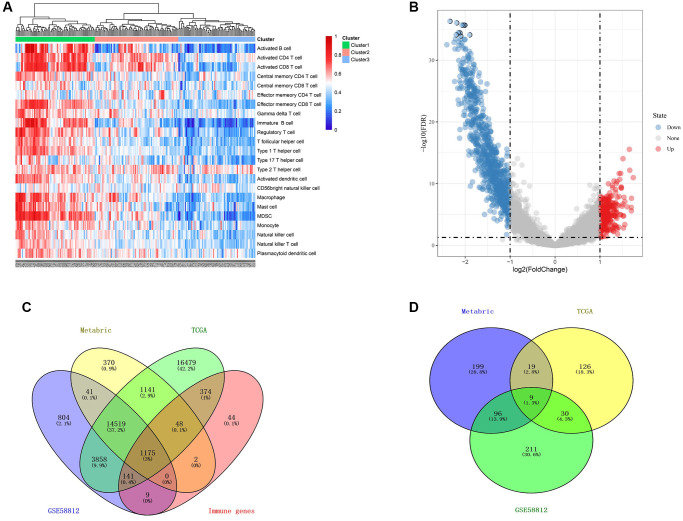
**Identification of gene signature related to immune in TNBC.** (**A**) immune related ssGSEA scores were estimated in the METABRIC cohort. (**B**) volcano plot demonstrated distinctive expressed immune-related genes between immune low and immune high groups (**C**) Venn diagram suggested 1175 immune related genes in the three cohorts. (**D**) Venn diagram suggested 9 prognostic hypoxia and immune related genes in the three cohorts.

Based on the ssGSEA scores that specified the abundance and efficacy of immune cell fractions, TNBC samples in the METABRIC cohort were hierarchically assembled in immune-high, -median and -low groups, which displayed distinct abundance and efficacy of immunocytes (Figure 3A). Then, immune-related DEGs were obtained via comparing gene expression in the immune-low group with those in the immune-high group (Figure 3B), 1793 promising candidates were identified and 1175 cross-cohort IRGs were extracted in the three datasets (Figure 3C).

### Construction of a hypoxia and immune-related gene signature for predicting prognosis

**Figure 4:**
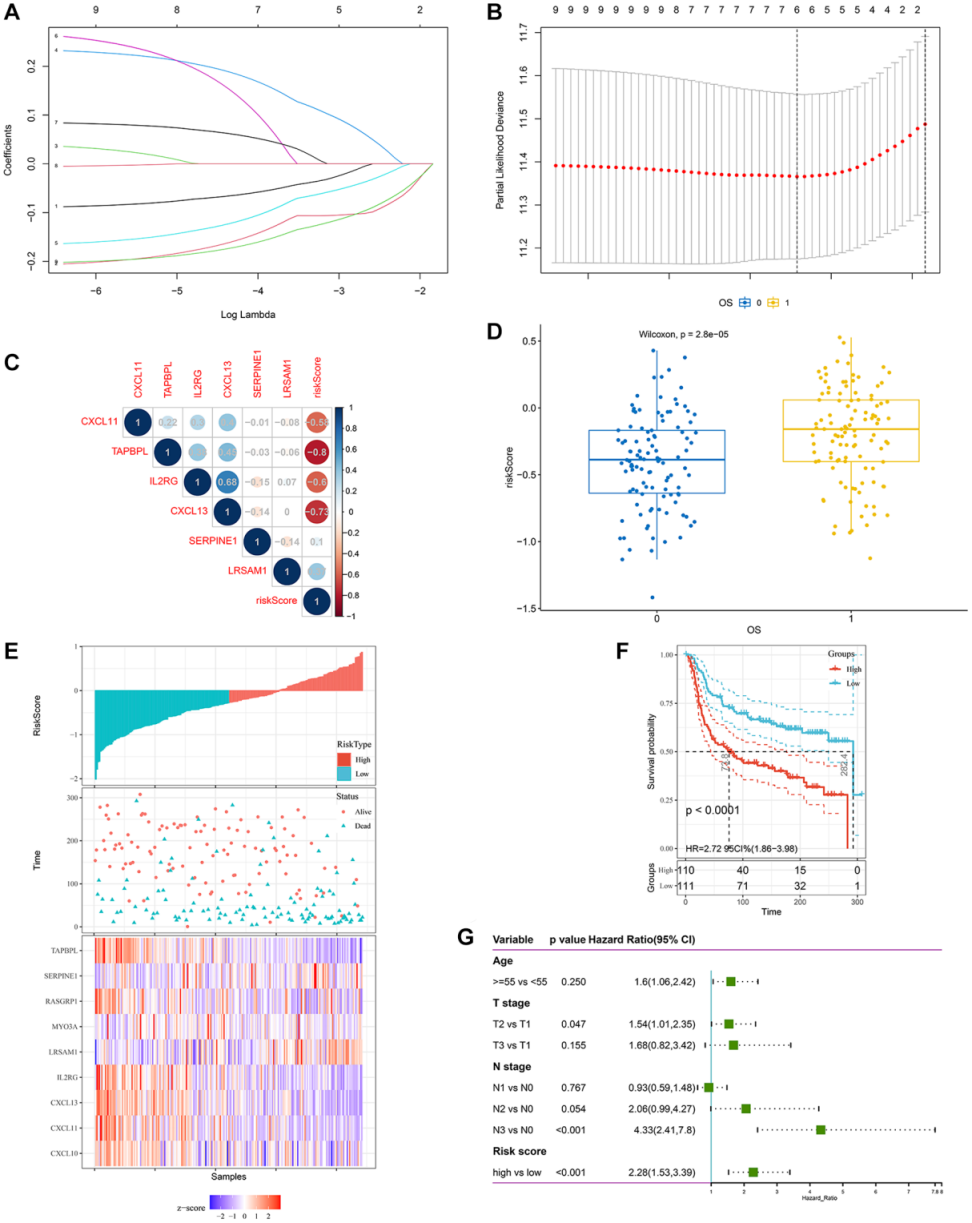
**Construction of a hypoxia and immune-related gene signature for prognosis.** (**A**, **B**) The LASSO coefficient profiles were constructed from 9 prognostic hypoxia and immune-related genes, and the tuning parameter (λ) was calculated based on the minimum criteria for OS with ten-fold cross validation. Six genes were selected according to the best fit profile. (**C**) Correlation between risk score and the selected 6 genes in the METABRIC cohort. (**D**) HIRS was remarkably increased in patients who died during follow-up. (**E**–**F**) Distributions of risk score, expression profile, and survival status of the gene signature. (**G**) Multivariate Cox regression model showed that HIRS as an independent risk factor for OS in the METABRIC cohort.

Based on the available 788 HRGs and 1175 IRGs, 9 cross-cohort prognostic genes were classified by performing the univariate Cox regression analysis (Figure 3D). Then, LASSO regression model was applied to select the most valuable markers for survival (Figure 4A). With the optimal log λ value of –3.36 generated, an ensemble of 6 genes (SERPINE1, IL2RG, CXCL11, CXCL13, LRSAM1, TAPBPL) remained with their distinctive LASSO coefficients (Figure 4B and Supplementary Table 1). Then, the selected genes were exerted to the formula above and HIRS was calculated in all cohorts. Spearman’s correlation test implied that HIRS was notably associated the selected genes (Figure 4C and Supplementary Figure 1A–1B).

### HIRS serves as a risk factor for prognosis in TNBC patients

Compared with alive patients, HIRS was remarkably increased in patients who dead during follow up in all datasets (Figure 4D and Supplementary Figure 1C–1D). Furthermore, worse prognosis has been exhibited in patients with higher HIRS than those with lower HIRS (Figure 4E–4F and Supplementary Figure 2A–2D). Among different clinicopathological parameters, multivariate Cox regression model showed that HIRS (HR = 2.28, *p* < 0.001) also as an independent risk factor for OSin the METABRIC cohort (Figure 4G). To validate the prognostic value of HIRS in external samples, it was further validated in the GSE103091 cohort. HIRS was remarkably increased in metastatic and dead patients (Supplementary Figure 3A, 3D). Distributions of risk score, expression profile, and survival status and of signature genes showed that patients with higher HIRS predicted worse MFS and OS than lower HIRS patients (Supplementary Figure 3B, 3C and 3E, 3F).

**Figure 5:**
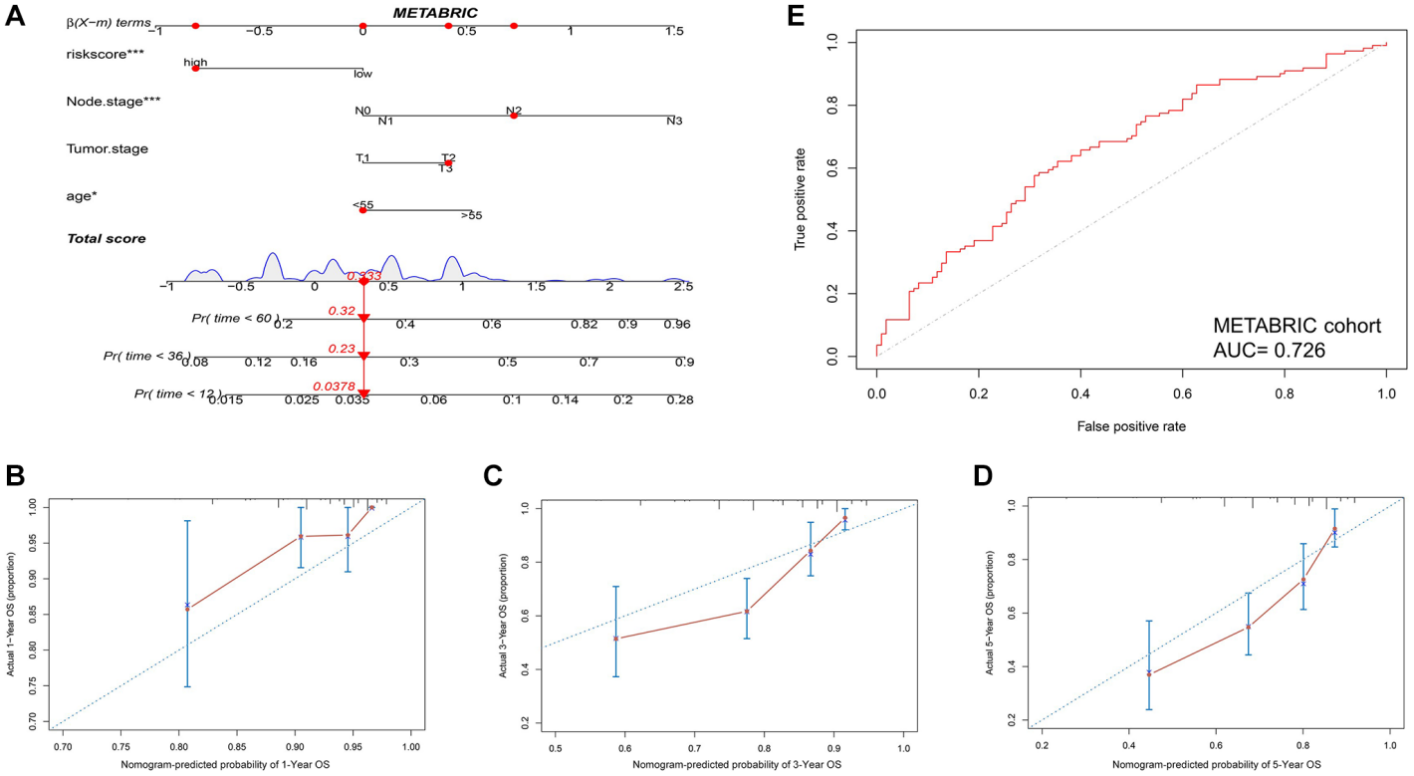
**Combination of HIRS and clinicopathological features optimize risk stratification and survival prediction in the METABRIC cohort.** (**A**) A nomogram was developed to analyze risk appraisal for individual patients. (**B**–**D**) Calibration analysis suggested a high accuracy of 1-, 3-, and 5-years OS prediction. (**E**) time-ROC analysis showed that the nomogram was a stable and reliable predictor for OS.

Then, a nomogram was constructed based on HIRS and other clinicopathological parameters in the METABRIC dataset to predict the survival for individual patients. As shown in Figure 5A, HIRS was an important predictor of OS in the visual model. Moreover, tROC and AUC were performed according to data availability (Figure 5B–5D and Supplementary Figure 4D–4F), the prediction accuracy of the nomogram for survival probability implied a promising predictive value of HIRS in the calibration analysis (Figure 5E and Supplementary Figure 4A–4C). Moreover, we analyzed the prediction accuracy of the nomogram in the GSE103091 cohort, the AUC values of the nomogram to predict 1-, 3- and 5-year MFS was 0.854, 0.759 and 0.711, respectively (Supplementary Figure 7A). The AUC values of the nomogram to predict 1-, 3- and 5-year OS was 0.860, 0.754 and 0.707, respectively (Supplementary Figure 7E). The calibration curves suggested that the nomogram-based predictive outcome had good consistency with the actual prognosis results (Supplementary Figure 7). The results revealed that the HIRS could be a promising marker for predicting clinical outcome in TNBC patients.

### Validation of hypoxia and immune profiling in HIRS

**Figure 6:**
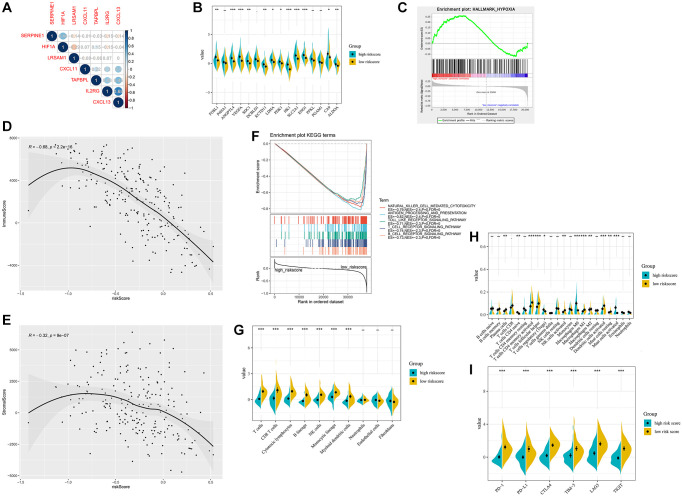
**Hypoxia-related sketch, immune-related sketch, and tumor infiltrating immune cells in the HIRS based groups in the METABRIC cohort.** (**A**) Correlation between the gene signature and HIF1A. (**B**) Correlation between HIRS and hypoxia-related genes. (**C**) GSEA confirmed the hypoxia status in the HIRS-based groups. (**D**) GSEA of immune-related signaling in the HIRS-based groups. (**E**–**F**) ESTIMATE analyses between different risk groups. (**G**) MCP-counter analyses between different risk groups. (**H**) CIBERSORT analyses between different risk groups. (**I**) the expression of immune checkpoint targets between different risk groups.

Next, we analyzed the correlation between the hypoxia-immune related gene signature and HIF1A. SERPINE1 was shown to positively correlate with the expression of HIF1A (Figure 6A and Supplementary Figure 5A, 5D). Besides, HIRS was significantly associated with hypoxia-related genes, implied that HIRS might reflect hypoxia status in the tumor microenvironment (Figure 6B and Supplementary Figure 5B, 5E). With the hypoxia gene set from MSigDB, GSEA analyses revealed that the association of hypoxia status and HIRS in the METABRIC dataset (Figure 6C). Furthermore, natural killer cell mediated cytotoxicity, toll-like receptor signaling pathway, antigen processing and presentation, T-cell activation, and B-cell activation were significantly enriched in the high-risk group according to the GSEA analyses (Figure 6D and Supplementary Figure 5C, 5F).

ESTIMATE algorithm indicated that HIRS was negatively correlated with the immune score in all cohorts (Figure 6E and Supplementary Figure 6A, 6F). Interestingly, an expressing reverse correlation between HIRS and the stromal score was also identified (Figure 6F and Supplementary Figure 6B, 6G). Meanwhile, MCP-counter suggested that patients with lower HIRS value had a higher level of tumor-infiltrating cytotoxic immune cells (Figure 6G and Supplementary Figure 6C, 6H). Furthermore, CIBERSORT algorithm (Figure 6H and Supplementary Figure 6D, 6I) confirmed that patients in the low-risk group were qualified with more antitumoral immune cells (plasma cells, activated dendritic cells, activated memory CD4 + T cells and NK cells), while patients in the high-risk group were characteristics of more regulatory T cells and M2 macrophages. Moreover, low-risk patients were correlated with a remarkably higher expression of immune checkpoint markers, like PD-1, PD-L1, CTLA-4, T-cell immunoglobulin and mucin-domain containing-3 (TIM-3), lymphocyte activation gene-3 (LAG3), and T Cell Immunoreceptor with Ig and ITIM Domains (TIGIT) comparative to that in the high-risk group (all *P* < 0.01) (Figure 6I and Supplementary Figure 6E, 6J).

### HIRS severs as a potential marker of therapeutic resistance

**Figure 7:**
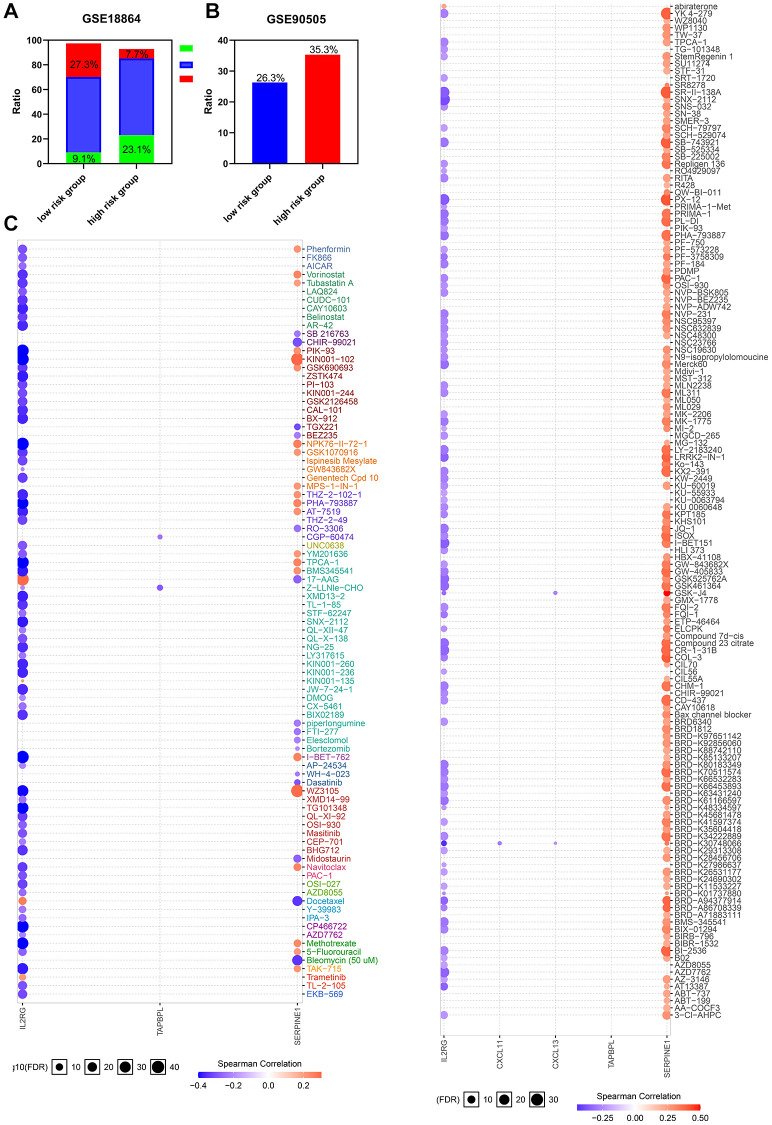
**The risk classifier serves as a favorable biomarker of resistance to chemotherapy.** (**A**) The ratio of complete response (CR) from GSE18864 cohort, and (**B**) the ratio of breast cancer related events from GSE90505 cohort in the HIRS based groups. (**C**) The relationship between gene signature and IC50 of different molecules in BRCA cell lines. PD, progressive disease; PR, partial remission, SD stable disease.

Considering tumor hypoxia and immune status always promote resistance to chemotherapy, whether the gene signature we constructed is a marker of therapeutic resistance needs further investigation. Patients from GSE90505 and GSE18864 were used to validate the prediction. As shown in Figure 7A–7B, the high risk group showed worse outcomes after chemotherapy in the GSE18864 and GSE90505 cohorts. Moreover, a landscape plot was explored by GSCALite to exhibit the relationship between drug reactions and the expression level of hypoxia-immune related genes (Figure 7C). The bubble heatmap depicted significant correlations between individual genes with IC50 data in BRCA cell lines. Thoroughly, SERPINE1 conferred drug resistance, while IL2RG exhibited drug sensitivity, which may help to explore targeted drugs to improve the clinical outcomes for TNBC patients.

### Validation for the indicative role of the HIRS in external cohort

**Figure 8:**
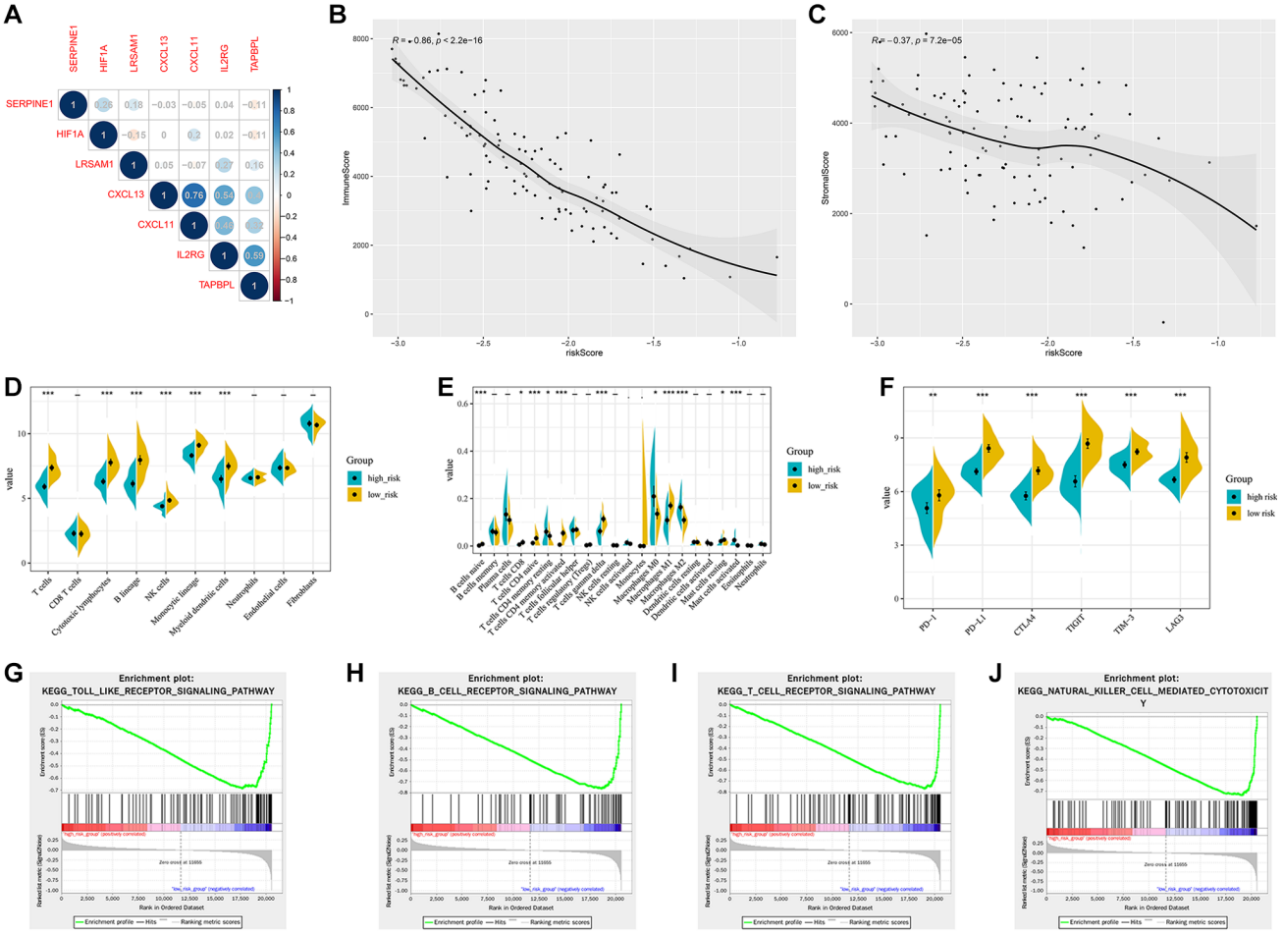
**Validation of the hypoxia and immune related gene signature in the GSE103091 cohort.** (**A**) Correlation network between the gene signature and HIF1A. Correlation between the risk score and immune score (**B**) and stromal score (**C**). (**D**) Association of MCP-counter-estimated infiltrating cells with the risk score. (**E**) Comparison of infiltrating immune cells (CIBERSORT) between different risk groups. (**F**) the expression of immune checkpoint targets between different risk groups. (**G**–**J**) GSEA of enriched immune-related signaling in the HIRS-based groups.

For the validation cohort, patients from GSE103091 were separated into different risk groups according to the median value of HIRS. Correlations of the gene signature with HIF1A expression were shown in Figure 8A. The immune and stromal scores were negatively associated with HIRS (Figure 8B, 8C). MCP-counter suggested that patients with lower HIRS value presented with a higher percentage of tumor-infiltrating cytotoxic immune cells (Figure 8D), and the CIBERSORT results showed that the high-risk group were qualified with more immune suppressive cells (Figure 8E). Meanwhile, patients with higher HIRS were exhibited significantly lower expression of PD-1, PD-L1, CTLA-4, LAG3, TIGIT, and TIM-3 comparative to those in the low- risk group (all *P* < 0.01) (Figure 8F). Furthermore, GSEA showed that immune related signaling were notably enriched in the high-risk group (Figure 8G–8J).

## DISCUSSION

To date, some hypoxia and immune related gene signatures for therapeutic response and prognosis have been established in different cancer types, like head and neck, gastric, breast cancer, and oral squamous cell carcinoma [37–40]. However, unavoidable deficiencies existed in previous studies. For instance, the hypoxia and immune-related gene signatures established in previous studies are roughly based on some public datasets or literature-reported genes, ignoring the fact that hypoxia and immune microenvironment as significant cancer hallmarks involving gene expression profiles.

Previous studies have addressed that hypoxia could reprogram the tumor microenvironment, resulting from the suppression of immune status in TNBC [16, 41, 42]. Given that hypoxia moderators and immune checkpoint inhibitors have been shown to present latent clinical application value in TNBC patients [41–44], we investigated the potential value of a combined hypoxia and immune gene signature for TNBC in this study. By using the ssGSEA and WGCNA methods, we evaluated the hypoxia and immune status in TNBC to choose the hypoxia related genes and immune related genes firstly, which promising the specificity and exclusivity of the gene signature we established in TNBC patients. Then, we selected 6 gene signatures that robust reflect the prognosis of TNBC patients using univariate cox regression and LASSO regression model.

Survival analyses demonstrated the six gene signatures were significantly associated with the prognosis of TNBC patients, and worse prognoses were observed in patients with higher HIRS. Moreover, the nomogram we constructed in this study had a favorable predictive performance for prognosis in TNBC patients. Both calibration plots and tROC curves indicated the stable and dependable performance of the nomogram for survival prediction in TNBC patients. As for hypoxia correlation, the key biomarkers of hypoxia (VEGFA, SLC2A1, ALDOA, ENO1CA9, etc.) are increasingly expressed in the high-risk group compared with their counterparts, implying a hypoxia status in the high-risk groups. Besides, GSEA analyses showed that patients from the high-risk group was significantly associated with hypoxia status.

For the association between the risk model with immune characteristics, Estimate analyses showed that both immune and stromal score were negatively correlated with HIRS, which indicated heterogeneous immune status within the different risk groups. In addition, MCP-counter demonstrated that activated immune cells including T cells, B cells and NK cells were sharply decreased for patients with high HIRS, implying an immune defect profile in this group. Besides, CIBERSORT revealed that patients with high HIRS had a remarkably higher percentage of M2 macrophages and Tregs phenotype. Whilst, immune-effective cells, like plasma cells, activated T cells and NK cells were decreased in the high-risk group, indicating that the risk model we constructed in this study may effectively predict the immune microenvironment. Immune checkpoints execute a vital role in carcinogenesis by espousing tumor immunosuppressive activities. Tumor cells can protect themselves from immune attack by activating immune checkpoint targets. Accumulating evidence suggested that patients with PD-L1 expression in tumor cells and stromal immune cells are more likely to respond to chemotherapy and immunotherapy and exhibit better prognosis [9, 45–47]. Previous study showed that hypoxia could induce high expression of PD-L1 on MDSCs and macrophages in tumor microenvironment, then suppresses the immune system to evade immune attack [48–50]. In our study, the expression of immune checkpoint markers was notably decreased in the high-risk group, which meant that the hypoxia-immune status in tumor microenvironment may affect the response to the immune checkpoint inhibitors (ICIs) therapy. Moreover, results from external cohorts and the GSCALite dataset indicated that the gene signatures we obtained could effectively depict the drug response of TNBC patients. Patients from the high-risk group showed worse outcomes after chemotherapy in GSE18864 and GSE90505 cohorts, which may explore targeted therapy to TNBC patients.

The comprehensive hypoxia–immune related gene signature we constructed indicated that personalized treatment should be exerted in distinct risk subgroups. For instance, immunotherapy, like anti-PD1/PD-L1 treatment, might be more effective in patients with low-risk feature. A lower HIRS was suggestive of a higher level of activated immune cells and higher efficacy for immunotherapy. Patients from the high-risk group had worse survival outcomes, a higher HIRS might indicate a hypoxia microenvironment, and limited probable benefit from immune checkpoint inhibitors. The findings indicated that further successive immunotherapy might effective after hypoxia modification.

Meanwhile, some limitations in this study should be noted. First, this is a cross-cohort and retrospective study, further verification in prospective trials is warranted. Second, the nomogram we developed need to be validated in external cohort to examine its performance and accuracy. Third, further experimental studies are needed to elucidate the underlying mechanism of the hypoxia and immune gene signatures in TNBC.

## CONCLUSIONS

In summary, a novel hypoxia and immune related gene signature to predict survival and discriminate high-risk patients with TNBC were developed and cross-cohort validated. The hypoxia and immune related risk model could be a powerful tool to select patients for hypoxia-targeted therapies and immunotherapies. Large-scale, multi-center and prospective studies are warranted to validate the efficacy of the HIRS model we constructed in the future.

## Supplementary Materials

Supplementary Figures

Supplementary Table 1
