# Supplementary material for: A combined hypoxia and immune gene signature for predicting survival and risk stratification in triple-negative breast cancer
Source: Aging (Albany NY). 2021 Aug 2;13(15):19486–509. doi: 10.18632/aging.203360 (PMC8386525; doi:10.18632/aging.203360)
Supplement: Supplementary Figures [file aging-13-203360-s001.pdf]

SUPPLEMENTARY FIGURES

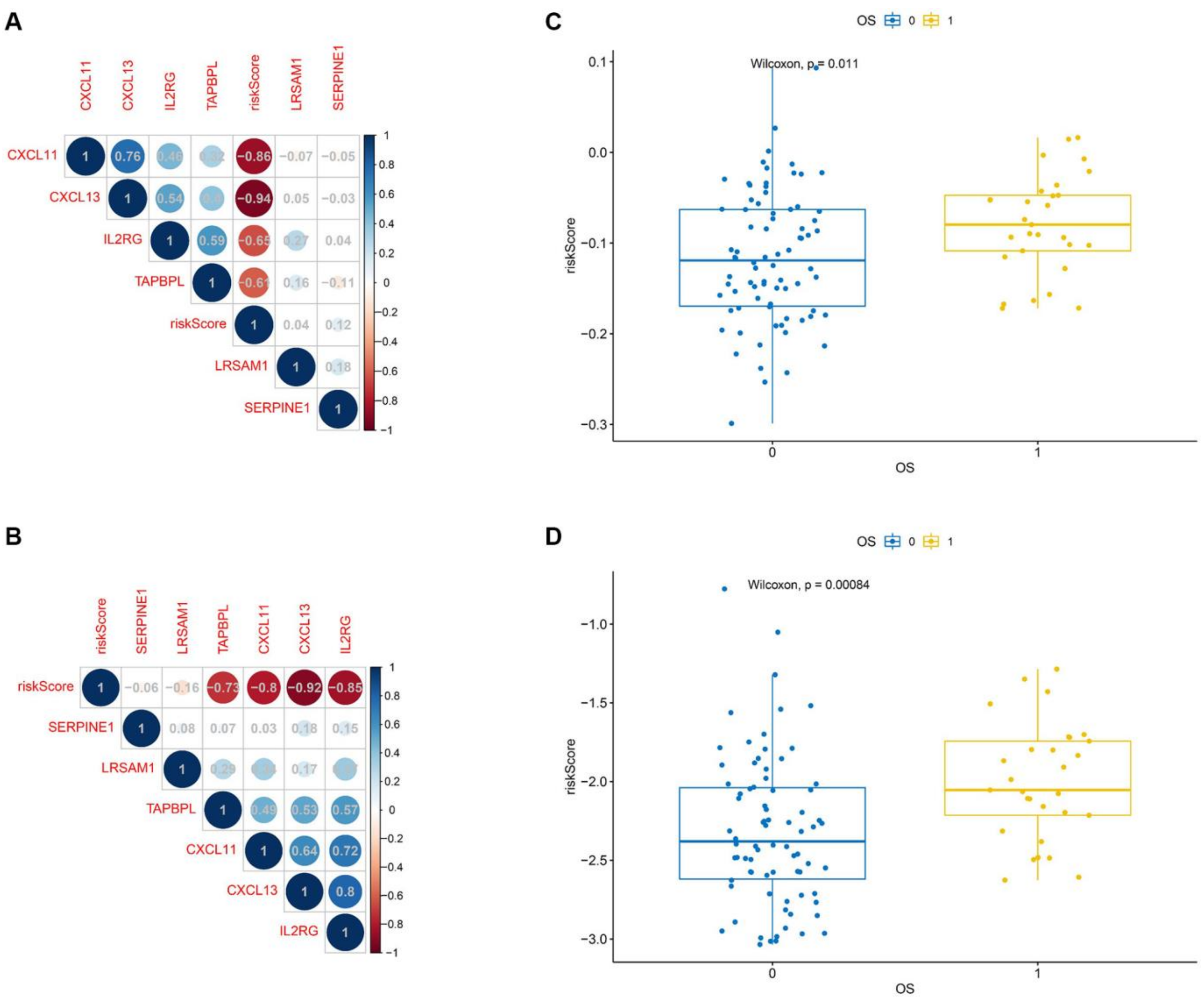

Supplementary Figure 1. Correlation between HIRS and the selected gene signatures in the TCGA (**A**) and GSE58812 cohorts (**B**). HIRS was remarkably increased in patients who died during follow-up in the TCGA (**C**) and GSE58812 cohorts (**D**).

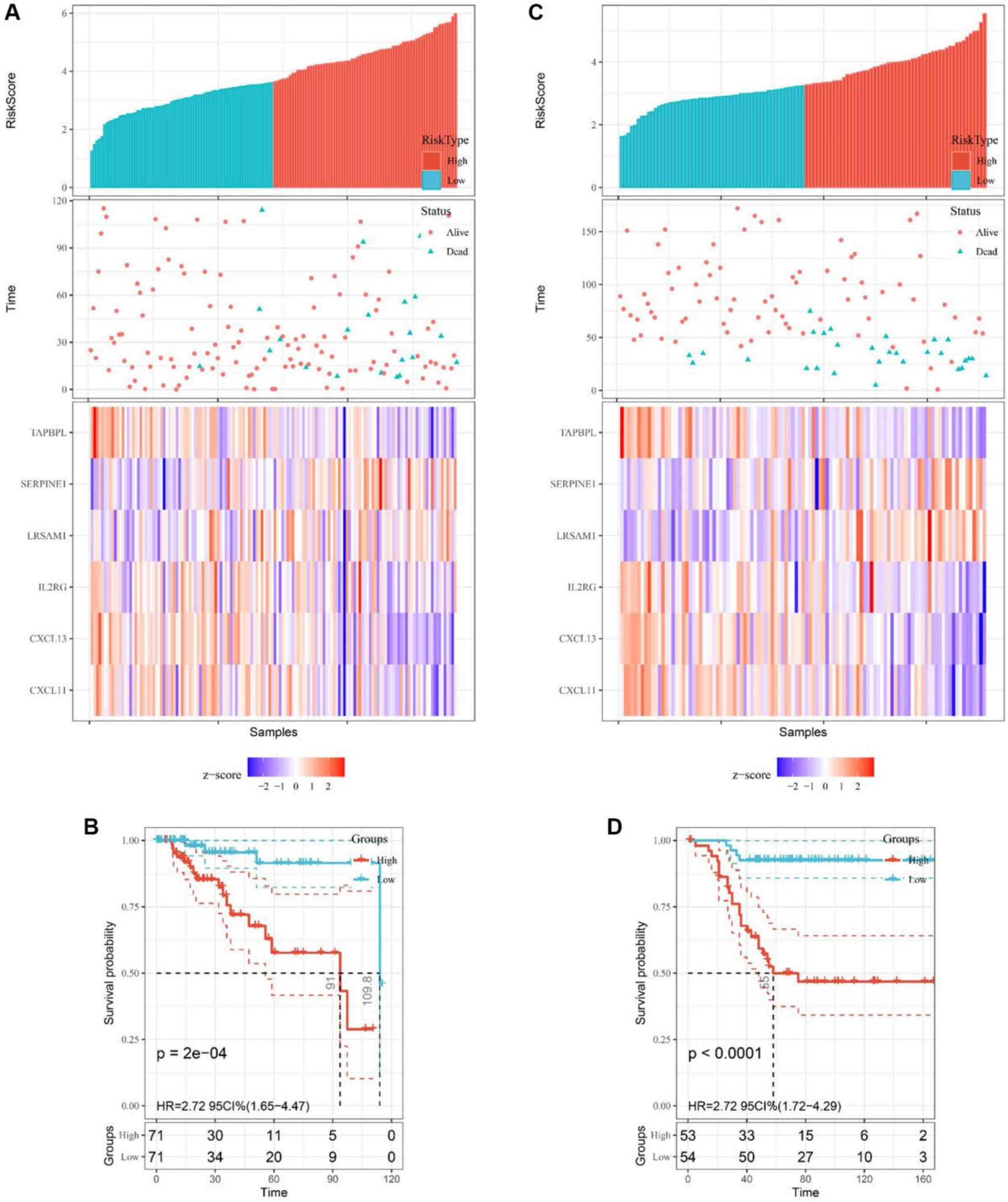

Supplementary Figure 2. Disposition risk score, expression profile, and survival status of gene signatures in the TCGA (**A**–**B**) and GSE58812 cohorts (**C**–**D**).

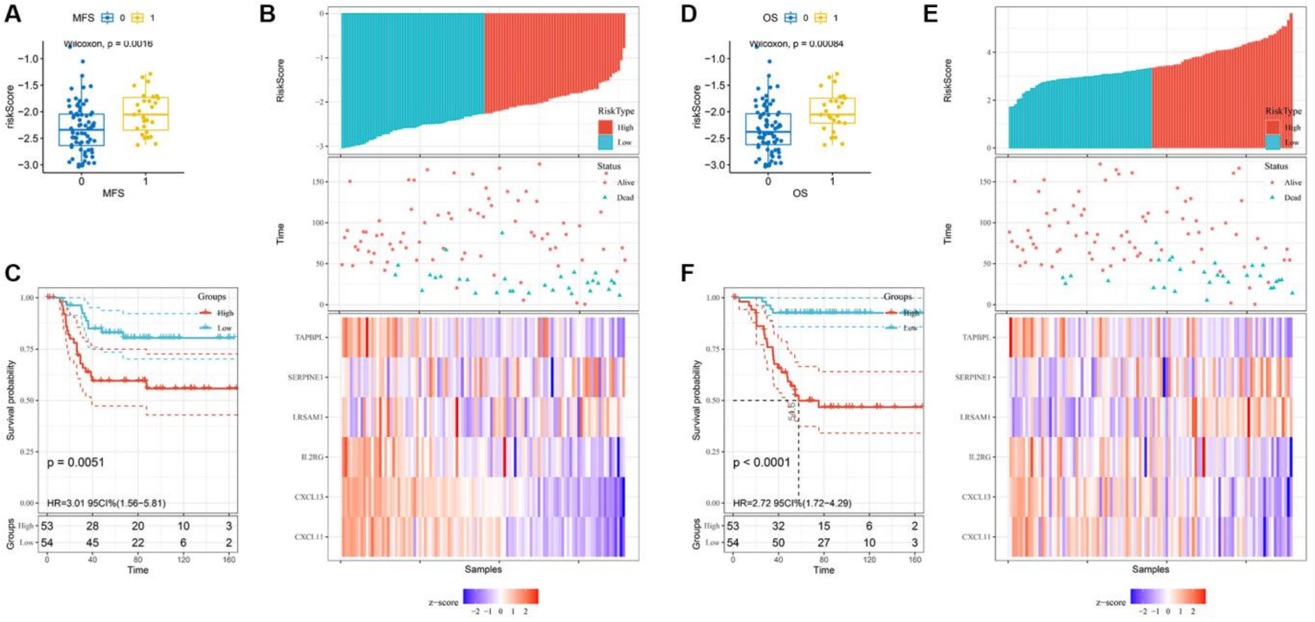

Supplementary Figure 3. Verification of the prognostic value of the hypoxia and immune gene signature in the GSE103091 cohort. HIRS was remarkably increased in patients who had metastases (**A**) and died (**D**) during follow-up. Distributions of risk score, expression feature of signature genes and MFS (**B**–**C**), and OS (**E**–**F**).

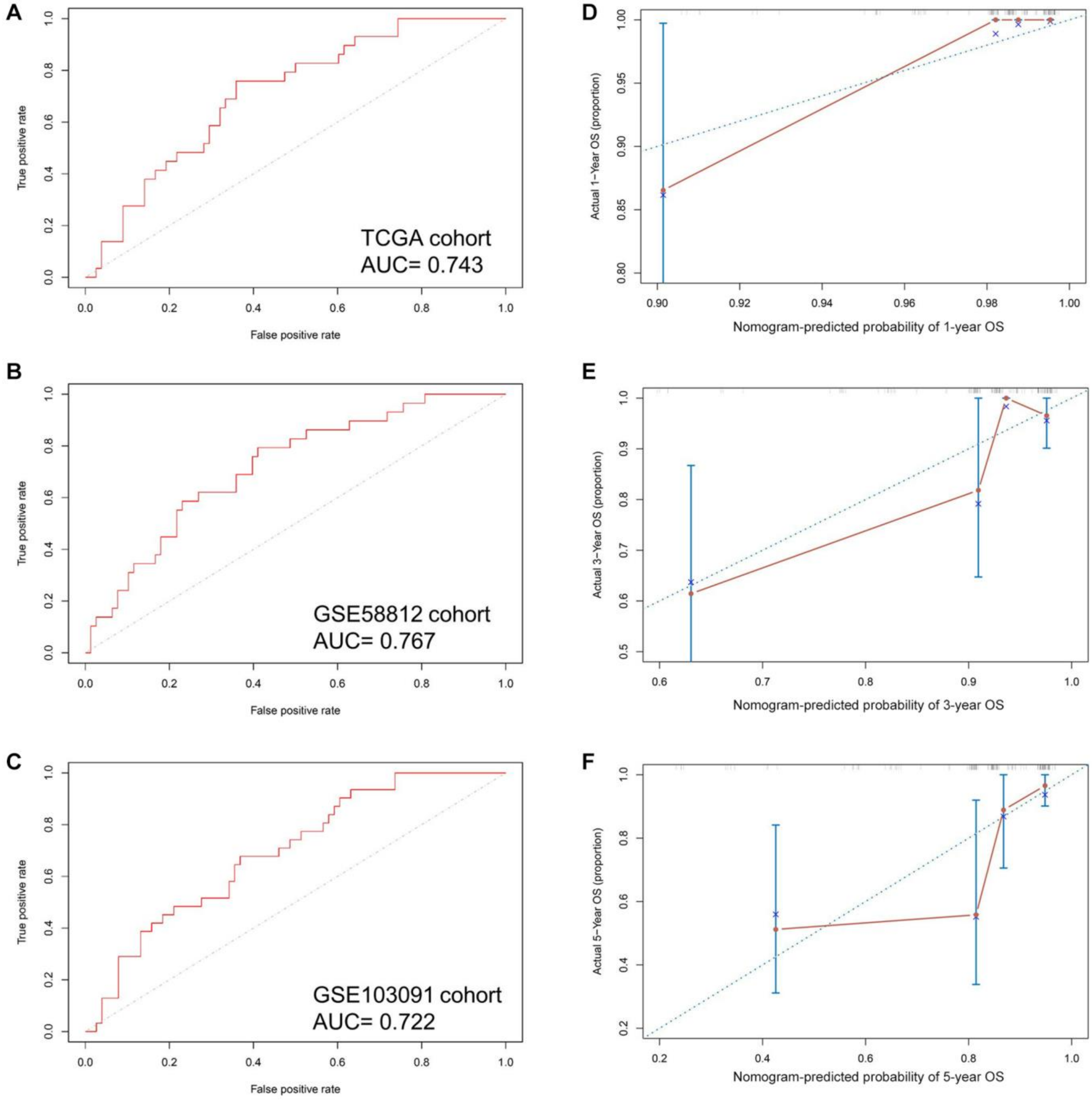

Supplementary Figure 4. Time-ROC analysis proved that the nomogram was a stable and reliable predictor for OS in the TCGA (**A**), GSE58812 (**B**), and GSE103091 (**C**). (**D**–**F**) Calibration analysis indicated a high accuracy of 1-, 3-, and 5-years OS prediction in the TCGA cohort.

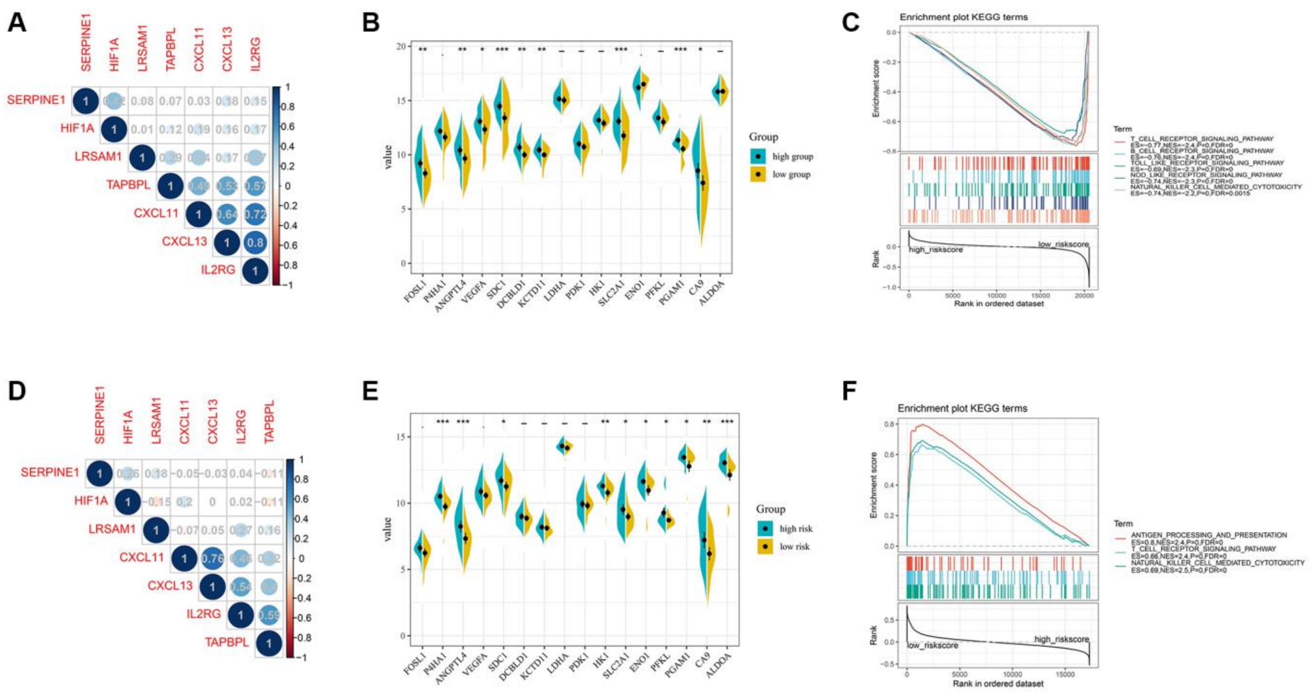

Supplementary Figure 5. Hypoxia and immune related profiling between distinct HIRS groups in the TCGA and GSE58812 cohorts. (**A**) Correlation between the gene signature and HIF1A in the TCGA (**A**) and GSE58812 (**D**) cohorts. Correlation between the risk score and hypoxia-related genes in the TCGA (**B**) and GSE58812 (**E**) cohorts. GSEA of immune-related signaling in distinct HIRS groups in the TCGA (**C**) and GSE58812 (**F**) cohorts.

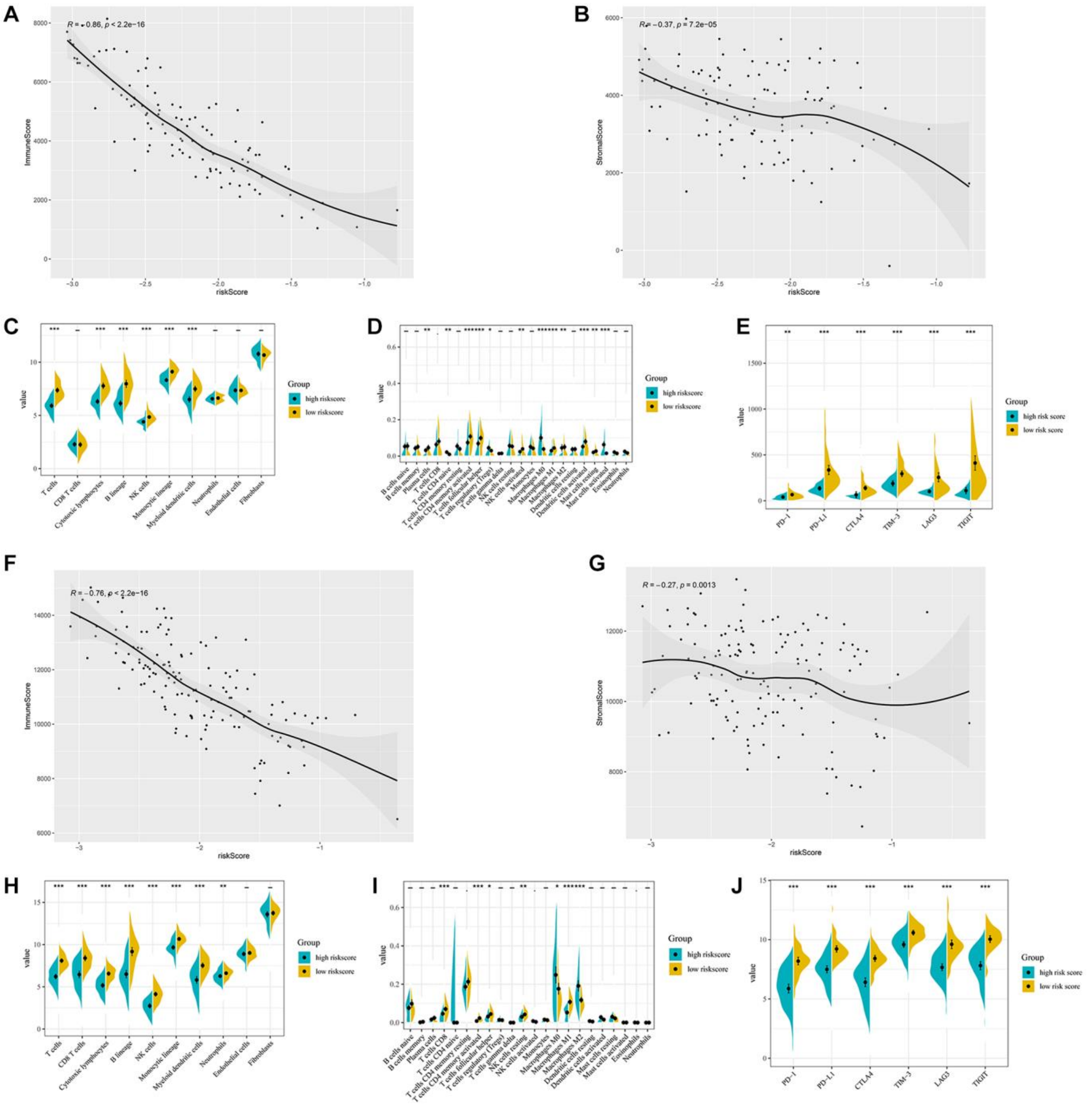

Supplementary Figure 6. Immune-related sketch, and tumor-infiltrating immune cells between distinct HIRS groups in the TCGA and GSE58812 cohorts. ESTIMATE analyses between distinct risk groups in the TCGA (**A**, **B**) and GSE58812 (**F**, **G**) cohorts. MCP-counter analyses between distinct risk groups in the TCGA (**C**) and GSE58812 (**H**) cohorts. CIBERSORT analyses between distinct risk groups in the TCGA (**D**) and GSE58812 (**I**) cohorts. The expression of immune checkpoint targets between distinct risk groups in the TCGA (**E**) and GSE58812 (**J**) cohorts.

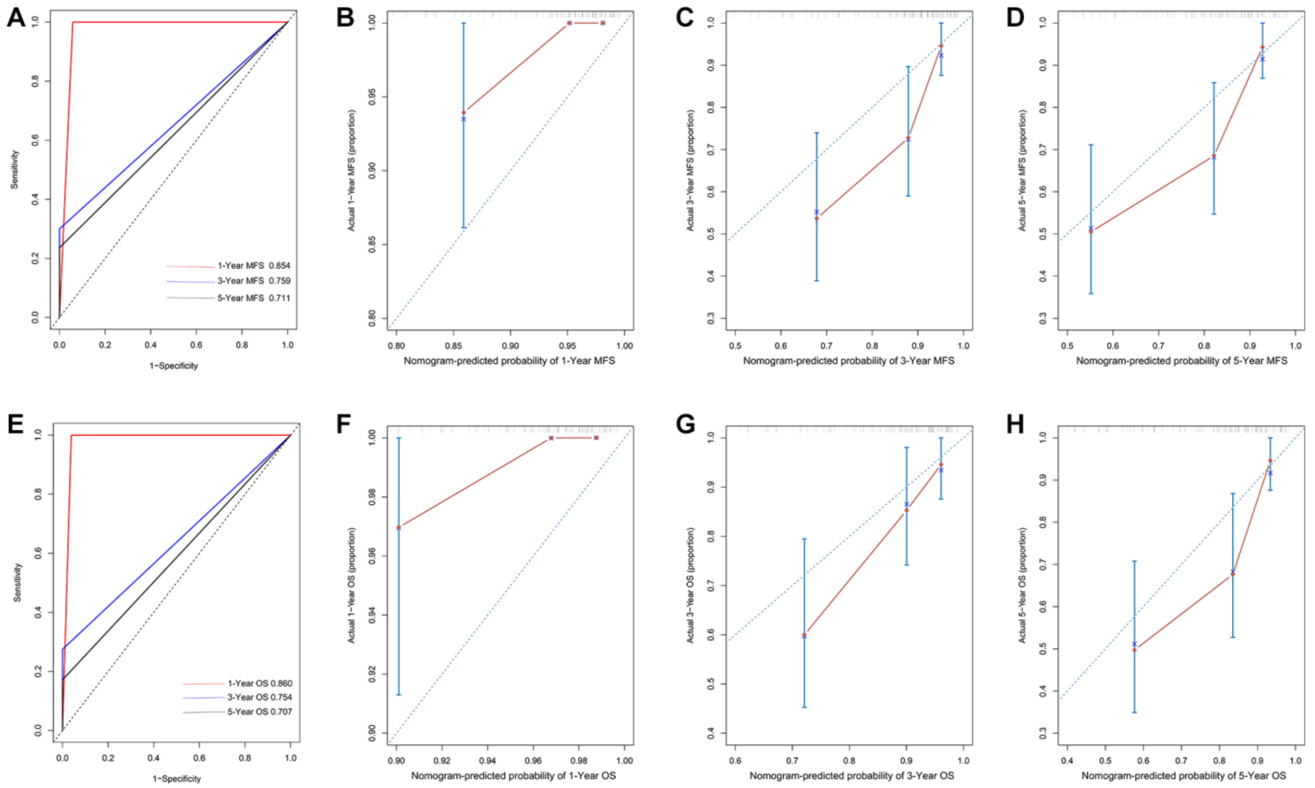

Supplementary Figure 7. Validate the prediction accuracy of the nomogram in the GSE103091 cohort. 1-, 3 -, and 5-years receiver operating characteristic curves for MFS (**A**) and OS (**E**). The calibration plots for predicting patient survival at 1-, 3- and 5-year point for MFS (**B**–**D**) and OS (**F**–**H**).
