# Supplementary material for: A combined hypoxia and immune gene signature for predicting survival and risk stratification in triple-negative breast cancer
Source: Aging (Albany NY). 2021 Aug 2;13(15):19486–509. doi: 10.18632/aging.203360 (PMC8386525; doi:10.18632/aging.203360)
Supplement: Supplementary Table 1 [file aging-13-203360-s002.pdf]

SUPPLEMENTARY TABLE

Supplementary Table 1. The distribution of LASSO coefficients of the gene signature.

Gene	Coef
IL2RG	–0.04006
CXCL13	–0.10619
LRSAM1	0.12198
CXCL11	–0.06781
SERPINE1	0.023619
TAPBPL	–0.13158
